# Cross-cultural differences in visuo-spatial processing and the culture-fairness of visuo-spatial intelligence tests: an integrative review and a model for matrices tasks

**DOI:** 10.1186/s41235-021-00350-w

**Published:** 2022-02-04

**Authors:** Corentin Gonthier

**Affiliations:** LP3C, University of Rennes, Campus Villejean, Place du Recteur Henri Le Moal, CS 24307, 35043 Rennes, France

**Keywords:** Raven's matrices, Visuo-spatial reasoning, Fluid intelligence, Culture-fair, Method bias, Cross-cultural psychology

## Abstract

Visuo-spatial reasoning tests, such as Raven's matrices, Cattell's culture-fair test, or various subtests of the Wechsler scales, are frequently used to estimate intelligence scores in the context of inter-racial comparisons. This has led to several high-profile works claiming that certain ethnic groups have lower intelligence than others, presumably due to genetic inferiority. This logic is predicated on the assumption that such visuo-spatial tests, because they are non-verbal, must be culture-fair: that their solution process does not significantly draw on factors that vary from one culture to the next. This assumption of culture-fairness is dubious at best and has been questioned by many authors. In this article, I review the substantial body of psychological and ethnographic literature which has demonstrated that the perception, manipulation and conceptualization of visuo-spatial information differs significantly across cultures, in a way that is relevant to intelligence tests. I then outline a model of how these inter-cultural differences can affect seven major steps of the solution process for Raven's matrices, with a brief discussion of other visuo-spatial reasoning tests. Overall, a number of cultural assumptions appear to be deeply ingrained in all visuo-spatial reasoning tests, to the extent that it disqualifies the view of such tests as intrinsically culture-fair and makes it impossible to draw clear-cut conclusions from average score differences between ethnic groups.

## Introduction

### Research on race and intelligence

Comparisons of average intelligence in different ethnic groups have flourished in psychology. Hundreds of studies have gone through the process of measuring reasoning performance in different groups, usually with a visuo-spatial test such as Raven's matrices (e.g., Raven & Raven, 2000); comparing their results; and drawing conclusions about their relative levels of intelligence. This has led to several high-profile syntheses (Herrnstein & Murray, 1994; Lynn & Vanhanen, 2002; Rushton & Jensen, 2005) reaching the conclusion that some ethnic groups demonstrate consistently lower intelligence than others. African countries, and persons of African descent, tend to fare particularly poorly.

These group comparisons have been repeatedly criticized on methodological grounds (e.g., Kamin, 2006; Neisser et al., 1996; Wicherts et al., 2010a, b). For example, some African samples with higher ability seem to have been excluded from inter-country comparisons without justification (see Wicherts et al., 2010a), not all samples are of a high quality (Lynn & Vanhanen, 2002, 2006, famously used average IQ at a school for the handicapped and brain-damaged in Spain as an estimate for average intelligence in Equatorial Guinea; see Kamin, 2006; Wicherts et al., 2010b), and some experiments might not have been quite neutral ("a certain awe and reverence which the native has for the white man ensured in every case at least a perfunctorily co-operative attitude"; Nissen et al., 1935). Critically, however, the basic point stands: "Blacks" score consistently lower than "Whites." Throughout the world, most ethnic groups do score consistently lower than Western subjects on visuo-spatial tests such as Raven's matrices (for a large-scale comparison, see Brouwers et al., 2009). This is a robust result and not a matter of debate (see Wicherts et al., 2010a). The actual issue is not about the existence of this score difference, but about its interpretation (e.g., Steele, 1997).

Researchers have often attributed these inter-group differences to genetically lower intelligence, leading them to compute correlations between intelligence and variables such as skin color (skin reflectance; Meisenberg, 2004) and amount of precipitation in a country (as an index of evolutionary history; Templer & Stephens, 2014). In *Thirty years of research on race differences in cognitive ability*, Rushton and Jensen (2005) conclude definitively that there is "some genetic component in Black–White differences in mean IQ." Of course, the basic issue with this conclusion is that ethnic groups do not only differ in terms of genes: they also differ in terms of culture. Thus, the claim that inter-group differences of intelligence are driven by genetic differences needs to establish that cultural differences do not play a role. Two major ways to do this have appeared in the literature.

The first way to assert the dominance of genes is statistical: draw on the tests' loadings on the *g* factor and their heritability coefficients. This tends to raise severe statistical problems (e.g., Schönemann, 1997a, b), but equally critical is the fact that these measures are heavily confounded with complexity and cultural load. In other words, tests with a higher *g*-loading are simultaneously more complex, more heritable, and more culturally loaded, which makes it impossible to disentangle the specific effect of genes (see Flynn, 2010; Gottfredson, 2016; Kan et al., 2013). The second way to assert that inter-racial differences are of genetic origin is to argue that the tests, usually visuo-spatial, employed to measure intelligence are culture-fair: in other words, that performance on these tests does not depend on culture to a significant extent. This assumption is the focus of the present paper.

### Visuo-spatial intelligence tests in cross-cultural comparisons

**Fig. 1:**
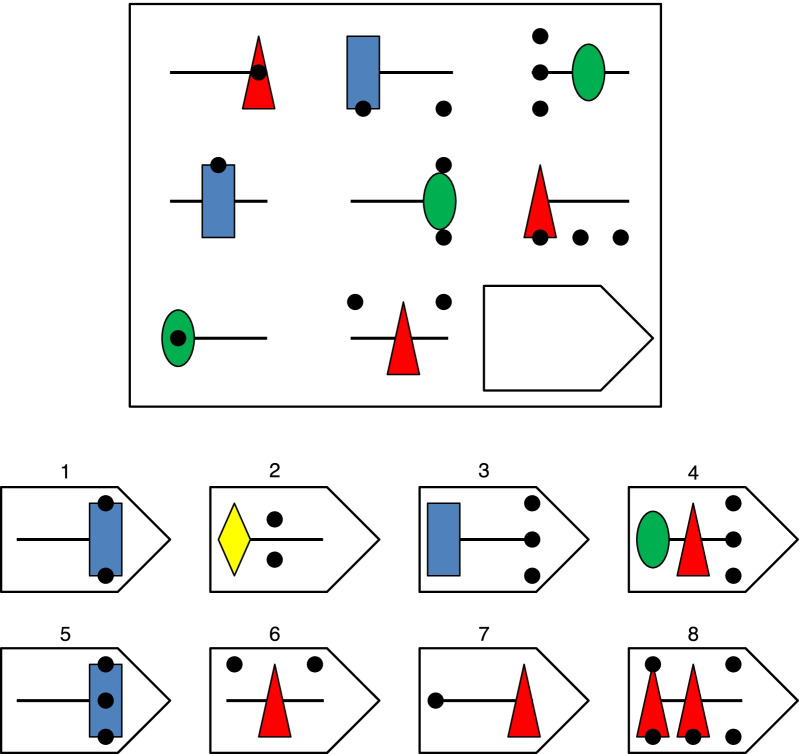
Example item for a matrix task. *Note* This example is fairly typical of what can be encountered in a matrix task. It is somewhat more difficult than most items in the versions of Raven's matrices usually employed for cross-cultural comparisons (due to more rules being included at the same time: distribution of three, movement, pairwise progression), and it uses more colors (but less than other versions, such as those of the Wechsler scales)

Most cross-cultural comparisons of intelligence have relied on visuo-spatial intelligence tests: tasks that require subjects to manipulate visual representations, usually of abstract geometric shapes and colors. The hallmark of this approach is the visual analogy test, of which a prime example is Raven's progressive matrices, which require subjects to understand the rules that connect abstract shapes arranged in a matrix so as to find a missing piece. An example item is displayed in Fig. 1. This is probably the test most frequently used in cross-cultural comparisons (Abdel-Khalek & Raven, 2000). Two versions of the test, Raven's Colored Progressive Matrices and the more difficult Standard Progressive Matrices have, together, contributed the bulk of cross-country datasets in syntheses such as Lynn and Vanhanen (2002, 2006). Subtests conceptually similar to Raven's matrices also appear in other batteries frequently used in this context, such as the Wechsler scales, the Kaufman-Assessment Battery for Children, and Cattell's Culture-Fair Test (see Wicherts et al., 2010b). Other visuo-spatial tests are also employed in cross-cultural research, though less frequently: examples include visuo-constructive tests, such as various versions of block design tests (e.g., Kohs' blocks, which require subjects to assemble colored blocks to recreate a drawn pattern), and mazes tests (e.g., Jahoda, 1956).

Raven's matrices and similar analogy tests are among the best measures of fluid intelligence, and they tend to demonstrate excellent psychometric properties, at least in educated samples (Carpenter et al., 1990; Raven & Raven, 2000). The greatest purported advantage of matrices tests for cross-cultural research, however, is that they are expected to be relatively unbiased by cultural differences (e.g., Jensen, 1974; Lynn & Vanhanen, 2002; Raven & Raven, 2000; Rushton & Jensen, 2005). Because visuo-spatial tests involve no verbal content except for the necessity of communicating instructions, and because the visual material they involve is limited to elementary shapes, performance on these tests is supposed to be independent from the culture of the subject.

The idea that non-verbal tests are less culturally biased than verbal tests is extremely pervasive in the literature. Critically, however, a number of studies have concluded the exact opposite: that there can be even greater cultural differences for visuo-spatial tests than for verbal tests (see Owen, 1998; Rosselli & Ardila, 2003). Multiple studies have shown higher performance on verbal than visuo-spatial intelligence tests, or even complete failure to perform the latter (for a discussion, see Rosselli & Ardila, 2003). Moreover, the psychometric properties of non-verbal tests may be significantly worse in populations that are culturally very distant from Western samples. A detailed review of the use of Raven's matrices in African samples (Wicherts et al., 2010a) showed that the test had a lower *g*-loading and lower convergent validity than in Western samples, that it demonstrated violations of unidimensionality, and that there was overall little support for its measurement invariance across cultures.

**Fig. 2:**
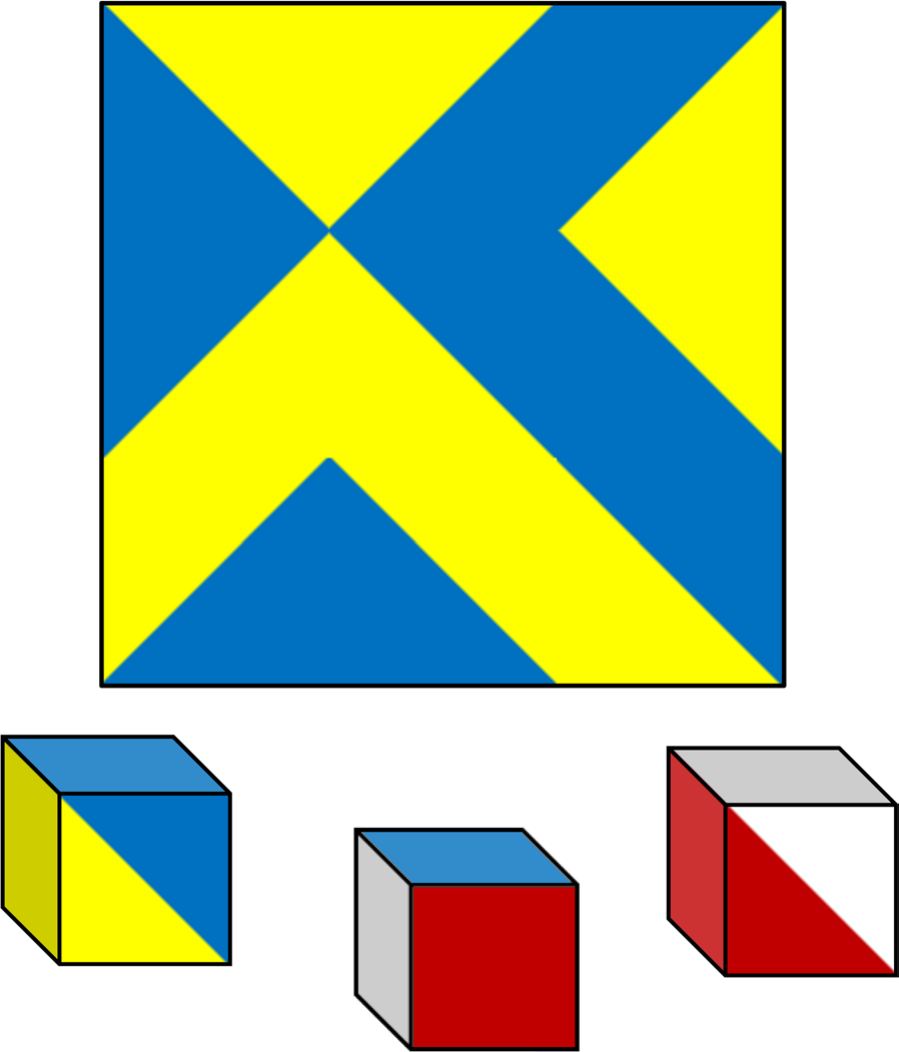
Example item for a block design test. *Note* This item is from the original version of Kohs' (1920) block design test. The target pattern, printed on a sheet of paper, has to be reproduced using cubes (whose sides are printed red, white, blue, yellow, red-and-white, and blue-and-yellow). The test is timed. Each design uses between 4 and 16 blocks (9 in the above example). Some designs are presented with the reference frame oriented as a diamond

The possibility of substantial cultural bias in non-verbal tests is by no means a novel discovery: this idea has long been defended by cross-cultural psychologists. For example, Biesheuvel (1951a) wrote that although "one generally has to fall back on pictorial or diagrammatic pencil and paper tests, or on performance tests involving form relations […] there are serious objections to the use of this material, the significance of which is far more dependent on culturally established habits than is commonly recognized. Sometimes one finds that the symbolism through which the test problems are stated is not understood, at other times that the skills required for their solution have not been equally developed in the cultures concerned." It will be useful here to relate a few examples to illustrate this point, drawing especially on block design tests (of which an example is given in Fig. 2). These visuo-constructive tests have the advantage of making differences in the solution process much more obvious than analogy tasks, on which the subject only points to a correct or an incorrect response which is then scored 0 or 1.

### Examples of results from visuo-spatial reasoning tests

McFie (1961) found that two different samples of Ugandan students (student nurses and technical students from the Bantu ethnic group) approached verbal tests in a way similar to English students, but that they had considerable difficulty in solving a test of block design or a test of reproduction of abstract designs from memory. Constructive performance was slow and preceded by a substantial period of exploration of the materials themselves; the reproduced patterns were often rotated or inverted, yielding very low scores. Performance was however adequate on verbal abstraction tests, such as the Similarities subtest of the Wechsler scale.

Jahoda (1956) found that a sample of teenagers in Ghana had trouble understanding a block design test, and had difficulty arranging the blocks: most subjects "clung persistently to a single block" at the outset, and the blocks were frequently placed in towers. Jahoda also noted that mazes were solved extremely slowly, much slower than by English-speaking subjects of comparatively lower ability. Raven's matrices elicited performance relatively close to an English sample, but only after considerable familiarization with the test.

Pontius (1989) found that a sample of Waodani adults in Ecuador had significant difficulty reproducing geometric figures, even using string instead of drawings. In a block design task, the subjects assembled blocks with complete disregard for colors and orientation: the four colors (red, blue, yellow, white) were selected arbitrarily and the designs were frequently rotated. Asymmetrical designs were frequently symmetrized by the subjects. Certain shapes were substituted with others, and the number of shapes in a design was frequently ignored (e.g., the subjects reproduced more triangles than were in the model). The author concluded that the subjects reproduced the global spatial features of the design, but that they ignored the precise relations between gestalts within the design. Similar results were obtained in another study (Pontius, 1995) involving Waodani adults and an additional sample of Dani and Asmat in Indonesia (West Papua). Subjects frequently answered that their productions were "the same" as the original design despite perceptually obvious differences.

Ardila and Moreno (2001), in an Aruaco sample in Columbia, also found very low performance on visuo-spatial tests: all participants were extremely slow, and multiple participants were unable to draw anything or arrange blocks at all. They were also incapable of drawing a map of the room they were in, and more generally of representing spatial relationships on paper. The authors labeled their three visuospatial tests as "frankly inappropriate." The subjects were able to complete verbal tests, as well as an ideomotor praxis test, pointing to a specific difficulty with abstract visuo-spatial materials more than with reasoning or spatial processing.

A few common trends emerge from these five studies, involving samples on three continents and ranging from barely-contacted Amerindians to nurse students. First, the subjects had specific difficulties with tests involving abstract visuo-spatial materials; they appeared otherwise capable of performing verbal tasks. In several instances, the subjects were baffled by the medium used for the test—cubes, drawing, pictorial representations; in others, they explicitly considered different shapes or different colors to be interchangeable, as confirmed verbally. The authors of these five studies all concluded that the inability of the subjects to perform adequately stemmed not from an innate difficulty in visuo-spatial reasoning, but from a lack of cultural expertise in perception, conceptualization or manipulation of this type of materials.

### Rationale for the review

Empirical evidence suggests that visuo-spatial intelligence tests can demonstrate substantial cultural bias, and degraded psychometric qualities, in populations that differ from Western samples. Theoretically, this makes sense: intelligence tests partly measure expertise in the manipulation of a certain type of material (Greenfield, 1998; Sternberg, 1999, 2004). A verbal test can be translated in the local language and communicated more or less appropriately with the help of an interpreter; but the structural medium of a visuo-spatial test is not translated and remains bound to the culture by which it was designed. This certainly plays a role in comparisons between countries and ethnic groups whose cultural uses of pictorial representations differ.

The common misconception that visuo-spatial intelligence tests such as Raven's matrices are culture-free tasks, rather than *a conventionalized cultural genre* (Greenfield, 1998), may be due to the lack of a clear overview of relevant literature. The current work attempts to remedy this problem by synthesizing two lines of research. In the next section, I review the various works that have demonstrated cultural variability in the processing of visuo-spatial materials, or at least those that are most relevant to visuo-spatial intelligence tests. (I leave aside cultural variation regarding more general aspects of the testing process, such as differences in the apprehension of timed performance, e.g., Agranovich, 2011). In the second part of the review, I outline the solution process of Raven's matrices and discuss how the various cultural differences in visuo-spatial processing listed in the first part can affect each step of that process.

This review is mostly focused on small-scale comparisons involving groups that are culturally distant from Western civilization (resembling the examples detailed above in “Examples of results from visuo-spatial reasoning tests” section). I devote little attention to the comparisons between very large samples that are more familiar to specialists of individual differences in cognition (e.g., Brouwers et al., 2009), because they tend to provide less detailed insight into the mechanisms that drive differences. I also devote little attention to comparisons between closer cultural groups within the same country. The major reason to focus on ethnographic data less familiar to psychologists (apart from the fact that remote samples are also included in large-scale comparison studies) is that many sources of cultural differences are not apparent when comparing groups that share close cultural practices. For example, the ability to recognize pictures as abstract representations of objects can contribute to the difference between remote African communities and Western countries, but its role will be invisible when comparing cultural minorities within the same Western country.

Critically, such comparisons are also very relevant to individual differences research in more familiar settings: biases exist on a continum. A difference in the mechanisms of parsing sequences of visual stimuli from left to right, or manipulating abstract visual shapes, may be more obvious in remote groups who read right to left and lack a name for triangles; but this difference can also have an effect in children from underprivileged ethnic minorities in a Western country, who will be less used to reading (which means their left-to-right scanpath may be less automatized), and less well-schooled (and thus less used to manipulating geometric shapes in abstract space). In other words, this is not a review of the cultural biases of visuo-spatial intelligence tests in remote cultural groups: this is a review of those processes involved in visuo-spatial intelligence tests that can be affected by cultural differences, as illustrated with the help of remote cultural groups, and as applicable to any and all cultural group comparisons.

## Cultural variability in the processing of visuo-spatial materials

The topics covered in this section are summarized in Table 1. General introductions to the topic of cross-cultural differences in visuo-spatial processing can be found in Deregowski (1989), Miller (1973), Serpell & Deregowski (1980), Levinson (1996), and Phillips (2019); see also Donoghue et al. (1978).

**Table 1:** Major sources of cultural differences in visuo-spatial processing applicable to visuo-spatial intelligence tests

Source of cultural variation	Examples
1 Understanding and interacting with pictures 1.1 Interaction with paper 1.2 Attention to the correct aspects of paper 1.3 Recognizing pictures as representations	Attention to surface features of the paper medium instead of the depicted information Lack of attention to visual information Difficulty in recognizing pictures as representations of real objects
2. Visual exploration 2.1 Horizontal bias as a function of reading direction 2.2 Other biases in visual exploration	Tendency to explore images in a direction consistent with the direction of reading Search for culturally relevant information Attentional capture by subjectively salient features of the display, such as color
3 Differences in analytic visual processing 3.1 Decomposition of visual gestalts 3.2 Conceptual or cognitive styles	Difficulty in decomposing gestalts into their component shapes, due to inexperience or to a cognitive style oriented toward holism
4 Perception and processing of visual objects 4.1 Geometric shapes 4.2 Colors 4.3 Numerosity 4.4. Size and distance	Difficulty in categorizing shapes, colors or numbers of objects as intended by the test designer due to different categorical names Difficulty in maintaining the identity of shapes, colors or numbers in memory due to the lack of corresponding names Difficulty with abstract manipulation of unfamiliar objects Less attention given to subjectively less important dimensions
5 Encoding of spatial relations 5.1 Use of a relative frame of reference 5.2 Encoding of other relations between objects	Different representation of relations between objects, with the item oriented as a function of cardinal points rather than the observer Difficulty in encoding relations such as "top," "bottom," "in" or "on" due to the lack of corresponding words
6 Understanding representations of movement and time 6.1 Movement and time in a single picture 6.2 Movement and time in a series of pictures	Difficulty in understanding that movement is represented in a picture, or conversely, erroneous perception that a picture represents a superposition of moments in time Difficulty in understanding causal and temporal relations between pictures
7 Understanding three-dimensional representations	Failure to recognize objects Misinterpretation of the background as being part of foreground objects
8 Symbolic meaning	Presence of unintended symbolic meanings in the display, leading to nameability or misinterpretation of logical relations
9 Response production	Lack of expertise with writing, drawing Lack of expertise with manipulating puzzles

### 1 Understanding and interacting with pictures

#### 1.1 Interaction with paper

The first necessary step of interacting with a visuo-spatial test presented on paper is recognizing the paper for what it is: a representational object that conveys information, and one that is not interesting in itself but only for the information that is printed on it. This is so obvious to Western readers that this topic is not usually discussed in cognitive models of visuo-spatial processing; but ethnographic data make it clear that this basic understanding of the paper medium is not at all obvious to people who have not encountered it before. Deregowski et al. (1972), in a study with Me'en in Ethiopia, found that when they were given pictures printed on paper, subjects would ignore the pictures and focus on the paper: they "felt the paper, sniffed it, crumpled it, and listened to the crackling noise it made; they nipped off little bits and chewed them to taste it" (Deregowski, 1989). The experience appeared stressful for some subjects; two tried to run away between presentation of the second and third picture. The subjects were at the same time capable of recognizing pictures printed on coarse cloth, and they became capable of recognizing the paper-printed pictures after gradual familiarization.

Utter lack of familiarity with the paper medium is one of the only biases reviewed here that can be expected to affect exclusively cultures very different from Western conceptions—trying to eat the test support is certainly an extreme case. It does however have the merit of drawing attention to the requirement of interacting with the test support itself, which is easily overlooked. This suggests the possibility of other, more subtle differences. For example, a related concept is that of sensotypes: it has been argued that African cultures place less emphasis on vision than on other senses, which could lead subjects to pay more attention to proprioception or auditory perception (Wober, 1966, 1967), to the detriment of systematic visual exploration of a test item. This could potentially contribute to differences in the analysis of visuo-spatial tests, though the idea is not consensual in the literature (for discussions, see Berry et al., 2002; Serpell, 1974; Witkin & Berry, 1975) and has remained largely speculative.

#### 1.2 Attention to the correct aspects of the paper medium

Visually examining a test item printed on paper does not guarantee that the testee will pay attention to the item: there are other things to look at on a sheet of paper, and they may be more interesting to an inexperienced observer than the item itself. Some subjects may be more interested in the white background and the white band around the drawing (Segall et al., 1966; see Miller, 1973), which can be particularly salient in an environment where white is rare. Other visual cues can also attract attention, such as the rectangular, thin and sharp edges of the paper sheet (Miller, 1973), its creases, and the sheen on the surface of the paper (Serpell & Deregowski, 1980). It seems that observers familiar with the paper medium have learned to ignore these cues to pay attention to the information itself (Serpell & Deregowski, 1980).

#### 1.3 Recognizing pictures as representations

There are many reports in the literature of the difficulty of people unaccustomed to Western modes of pictorial representation in recognizing that pictures depict *something* (see Deregowski, 1989). Evidence comes from numerous countries in Africa (such as studies in Nigeria and with the Kpelle in Liberia), but also from other cultures such as the Ainu in Japan (Deregowski, 1989). Herskovits (1950) cites the example of a mother incapable of recognizing a photograph of her son (for this and other examples, see Deregowski et al., 1972; Miller, 1973; Serpell & Deregowski, 1980). Studies have often found subjects incapable of recognizing pictures even of objects that are familiar to them (such as huts or pots; Biesheuvel, 1951b). The problem can be expected to be worse when objects are unfamiliar, or when the context provides too much or too little information (Miller, 1973). Note that this is not limited to paper: the understanding of representation can be equally difficult for puzzles (Biesheuvel, 1951b; Nissen et al., 1935) or drawings using strings (Pontius, 1989).

Understanding that a two-dimensional arrangement of shapes and colors can represent a real-life object seems to be an insight phenomenon (Miller, 1973): a sudden change in perception that occurs when the observer understands that information is being conveyed ("*oooooooh.* And in the space of 30 sec. the task was completed without error": Nissen et al. (1935); "Such representation was a completely amazing novel experience to the Aucas […] expressing utter amazement and delight in a most stirring way, as the wonder of representation began to dawn in their minds": Pontius, 1989).

### 2 Visual exploration

#### 2.1 Horizontal bias as a function of reading direction

Not all languages read left-to-right: others, such as Hebrew or Arabic, read in the right-to-left direction. These reading habits have a well-known effect on exploration of visual materials: readers of left-to-right languages tend to scan visual images from left to right, and the reverse is true for readers or right-to-left languages (Abed, 1991; Keenan, 1972). Languages with inconsistent reading direction, such as Japanese, are associated with less pronounced bias (Abed, 1991; Nachshon & Hatta, 2001). This is true even for abstract stimuli that have no intrinsic directionality (Harcum & Friedman, 1963; Nachshon et al., 1977).

The leftwards or rightwards bias in visual exploration extends to equivalent biases in line bisection (Chokron & De Agostini, 1995; Chokron & Imbert, 1993), the mental representation of actions (Dobel et al., 2007), the execution of spatially directed movements (such as drawing a circle clockwise or counterclockwise; Fagard & Dahmen, 2003), and aesthetic preference (Chokron & De Agostini, 2000). It biases even relatively low-level mechanisms, such as inhibition of return (Spalek & Hammad, 2005) and the shape of the perceptual span (Paterson et al., 2014). Subjects may also have an easier time manipulating and remembering information that is structured with a spatial flow consistent with their leftwards or rightwards directional bias (McCrink & Shaki, 2016).

This pervasive directional bias can be expected to affect exploration of any visuo-spatial material, so that speakers of a right-to-left language will analyze visual scenes in the reverse direction. It is also worth mentioning here the case of illiterate subjects, or subjects whose culture has no writing system at all. It can be expected that the visual exploration of these subjects will be less often in the usual direction (for an example, see Ardila et al., 1989), but also less systematic and less horizontally structured. Reading from top to bottom, as expected by the designers of tests such as Raven's matrices, may also not be obvious to inexperienced readers (see also Brouwer, 1995).

#### 2.2 Other biases in visual exploration

The horizontal directional bias has been the most documented, but culture may also influence visual search in other ways. An example is the faster search for long lines among short lines than the opposite in North American speakers, and the absence of this search asymmetry in Japanese speakers, possibly due to differences in orthographical systems (Ueda et al., 2018). Such fine-grained differences require accurate measurement of a type that has not often been used in cross-cultural studies, and they have only recently begun to appear in the literature. More generally, visual exploration of a scene is influenced by low-level features of the stimuli such as texture and luminance (Kollmorgen et al., 2010), and it could well be the case that what stimuli appear to be salient vary as a function of culture and the specificities of the everyday environment (for similar ideas, see Segall et al., 1963).

At a low level of abstraction, there seems to be a marked preference for color over shape in multiple African cultures, especially in the case of abstract designs (for details, see Serpell, 1974), which could make colored elements relatively more salient for observers (Brouwer, 1995). At a higher level, visual exploration may depend on the observer's expectations and preferences, and on what they consider to be salient and important in a scene. The scanpath, i.e., the visual path a subject follows to analyze a scene, appears to be both dependent on the observer's purpose (DeAngelus & Pelz, 2009; Yarbus, 1967) and highly idiosyncratic for a given subject (Noton & Stark, 1971). This could conceivably be accompanied with cross-cultural differences in the analysis of complex scenes. For example, Brouwer (1995) reports observers focusing on the appearance of a woman in a picture, and commenting that this woman wore different clothes and a different hairstyle from people in their neighborhood. More generally, Brouwer (1995) notes that Western observers are used to "take as a starting point that the thing in the foreground is the most important," whereas viewers from rural Africa "start with the thing that attracts their attention first."

### 3 Differences in analytic visual processing

#### 3.1 Decomposition of visual gestalts

Multiple authors have commented on the finding that subjects from African cultures tended not to decompose complex visual gestalts into their component shapes, and to treat them as undifferentiated whole (e.g., Biesheuvel, 1951b; this is not always found, however, e.g., Davidoff et al., 2008a). Cryns (1962) provided examples in a few samples and commented that all reveal "the same trend: the African’s inability to transcend the syncretic form of a perceptual Gestalt." Similar results have appeared in studies using visuo-constructive tasks involving other populations (see “Examples of results from visuo-spatial reasoning tests” section): for example, Pontius (1989, 1995) concluded that Waodani subjects in Ecuador and Dani and Asmat subjects in Indonesia focused on the global features of the pattern they had to reproduce, while paying little attention to the intra-pattern details, frequently making changes to the number, size or orientation of component shapes.

Cryns, however, may be a little too quick in concluding that this is an "inability" on the part of subjects to decompose the gestalt: it may simply be the case that these subjects have no cultural notion that intra-pattern differences are significant. On that note, Pontius (1995) commented that "Hunter-gatherers' survival depends on prompt assessment of the salient shapes of prey and attackers. By contrast, literacy skills require painstaking assessment of subtle intrapattern spatial relations among shapes." There is some converging evidence that focusing on global versus local information can be modulated by the environment people live in, such as degree of urbanization (Caparos et al., 2012). The difference is also sometimes found in the opposite direction: one study found that Maori men had a greater tendency than Western subjects to decompose a complex figure into its constituent elements in a copy task (Ogden & McFarlane-Nathan, 1997).

The tendency not to decompose visual gestalts can appear in visuo-spatial tests as a tendency to transform stimuli in the direction of a "good shape," with a dominance of holistic properties (see Wagemans et al., 2012). Examples of such transformations are the frequent rotations of patterns in block design tasks by subjects from non-Western cultures (Deregowski, 1972; Jahoda, 1956, 1976, 1978; McFie, 1961; Nissen et al., 1935; Pontius, 1989, 1995; Shapiro, 1960). Critically, these rotations are not random and appear to bear a relation with horizontality (Jahoda, 1976). In general, the designs tend to be rotated so that they are horizontal, stable: with their sides parallel to the edges of the reference frame, avoiding diagonal lines (Deregowski, 1972, 1989; Nissen et al., 1935; Shapiro, 1960). Critically, these rotated patterns are often considered by the subjects to be "the same" as the target design, confirming that this is not a constructive difficulty but a lack of importance given to orientation (Jahoda, 1978; Pontius, 1995).

It is noteworthy that subjects do not always align their designs horizontally in relation to the reference frame. Indeed, several authors have commented that their subjects ignored orientation as long as the overall shape was correct—or, perhaps, oriented their productions in the direction they were facing at the time of creation rather than in the direction of the reference frame (Biesheuvel, 1951b; Hudson, 1967). This is usually counted as an error in block design tests. On that note, Miller (1973) commented that treating the bottom edge of a paper as a base-line reference is probably a learned convention that does not transcend cultures.

At least two related types of transformations, where the global shape has precedence over local features, regularly appear in the literature. One is symmetrization errors, where deviations from vertical symmetry are ignored in reproducing a design (Jahoda, 1976; Pontius, 1989), and which may be related to the relative rarity of asymmetrical shapes in nature. The other is inversion errors, where designs are reproduced as a mirror image of themselves (Biesheuvel, 1951b; McFie, 1961). Again, such errors are not random and can reflect different emphasis on the importance of global over local features (Pontius, 1995). A related case is categorical perception of mirror images. Observers in some cultures (Mayan and some Tamil communities) consider left–right reflections of the same object as identical, even when instructed to treat them differently (Danziger & Pederson, 1998; Pederson et al., 1998). This could be related to the development of literacy, which in many languages requires children to learn that "b" and "d" have different meanings (Danziger & Pederson, 1998), though it might also have to do with the lack of a relative frame of reference in coding spatial position (see “Use of a relative frame of reference” section).

#### 3.2 Conceptual or cognitive styles

The tendency not to decompose gestalts into their component elements has often been described, not as a deficiency on the part of the subjects, but as a particular cognitive style, driven by culture: a preferential way to perceive and process information. In this view, Western cultures would be oriented toward analysis of the details of a visual display, whereas other cultures would be oriented toward perception of the globality of the display. This opposition has been variously described as a contrast between analytic and relational conceptual styles (Cohen, 1969), between analytic and holistic perception (Nisbett & Miyamoto, 2005), or between field independence and field dependence (Witkin & Berry, 1975).

Most cross-cultural research was performed within the field dependence/independence framework, with the idea that Western peoples tend to be more field independent—more focused on the parts than on the whole. This approach was notably defended by Berry (1976; for discussions, see Dasen & Mishra, 2013; Witkin & Berry, 1975), who argued that the preferred cognitive style within a culture was related to its ecological context. Berry concluded that cultures that rely on agriculture, such as the Temne people in Sierra Leone, tended to be more field dependent than cultures that rely on hunting or gathering, such as the Eskimo; this conclusion was replicated in numerous studies (see Witkin & Berry, 1975), and this difference was found to depend on acculturation (Dasen & Mishra, 2013).

The idea that cultures differ in cognitive styles has also emerged in the analytic-relational framework proposed by Cohen (1969), and in a series of studies comparing Western and Eastern cultures (Kitayama et al., 2003; Miyamoto et al., 2006; Nisbett & Miyamoto, 2005). The latter studies concluded that people from Asian cultures had a tendency to pay more attention to contextual and relational information than Western samples and to be more influenced by context, even in relatively low-level perceptual tasks (e.g., Doherty et al., 2008; Kitayama et al., 2003). This has been variously attributed to the existence of a more collectivistic mindset (see Dasen & Mishra, 2013) or to the greater cluttering of everyday visual scenes in Asia (Miyamoto et al., 2006).

### 4 Perception and processing of visual objects

#### 4.1 Geometric shapes

Abstract geometric shapes, such as circles or squares, straight or curved lines, form the basis of most visuo-spatial intelligence tests. These shapes are often supposed to be innate, culture-free categories, but this is not the case (see Owen, 1998). For example, infants do not show a preference for regular over irregular geometric shapes (Bomba & Siqueland, 1983), they do not necessarily create abstract categories such as "straight" versus "curved" (Abecassis et al., 2001) and they do not necessarily categorize objects as a function of shape (Abecassis et al., 2001; Smith, 1989). Culture has at least two effects on processing of geometric shapes. The first concerns the availability of words in the language to refer to a given shape, and the corresponding effects on cognition—categorization, memory, etc. The second concerns the relative familiarity of members of a given culture, or a given ecological context, with certain geometric shapes.

Regarding words, many authors have commented on the lack of names for geometric shapes in a number of cultures. At the time when such studies were published, the Himba language of Namibia had no words for circles, squares or triangles (Roberson et al., 2002). Swahili spoken by the Bantu in South Africa had no words for the concepts of triangles or squares (Ombredane, 1951; Serpell, 1974); they had no specific word for a circle and used the same "round" word for all sorts of circles and ellipses (Myambo, 1972). The Temne in Sierra Leone distinguished straight from curved lines but used the same word for squares, rectangles and cubes, and a different word for circles; these shapes were not strictly defined (Littlejohn, 1963). The Kpelle had names for some shapes, but these were used to refer to shape-like concepts more than strictly defined geometric shapes: the word "circle" was used for pots, frogs, sledgehammers, and turtles, basically any object with a closed shape and a modicum of circularity; the word "triangle" was used for arrowheads, tortoise shells and bird's nests (Cole et al., 1974). The sole cultural universal in shape description, if there is one, may be extendedness—references to length, width, and the length–width ratio (see Willats, 1992; Roberson et al., 2002). Note that even when names exist in a given language, the tested subjects do not necessarily know them, which has the same practical implications. Rural children in Zaire in one study could not name simple geometric shapes at all (Boivin et al., 1995).

The lack of words, or their imprecision, can structure cognitive activity to an extent: this is the linguistic relativity hypothesis outlined by Sapir and Whorf (see e.g., Kay & Kempton, 1984; Lucy, 1997). Names affect categorization: shapes that have the same name will tend to be grouped together, and conversely, subjects will have difficulty identifying an abstract geometric concept for which they do not have a precise name (for a demonstration, see Cole et al., 1974, who also found that this difficulty decreased with formal schooling). Languages that place less emphasis on shape also elicit less perceptual grouping by shape (see Lucy & Gaskins, 2001; Serpell, 1974).

Names also affect memory: having a specific name for a specific shape facilitates learning; when geometric shapes do not have names, there is no advantage for learning with regular rather than irregular shapes (Roberson et al., 2002). Of course, this fits with the notion of chunking in memory. A name can serve to compress information in memorizing visuo-spatial information: it dispenses the observer from having to separately encode enough visuo-spatial features to recreate the perceptual configuration (see e.g., Gonthier, 2020). Note that this is not just a question of having a name in the language or not: names that are shorter or have simpler syllabic structures are easier to rehearse and can thus elicit better memory (e.g., Chincotta & Underwood, 1997; Ishikawa & Nobe, 1998), which is another potential source of cross-cultural differences.

A second expected effect of culture on processing of geometric shapes is related to familiarity, which includes at least two questions: how familiar is a given shape in a given culture? How does expertise in manipulating abstract shapes facilitate their processing? As for the first question, a number of researchers have provided evidence for an effect of everyday familiarity on perceptual processing, especially in the context of visual illusions (for reviews, see Serpell, 1974; Deregowski, 1989; Berry et al., 2002; for another example, see Davidoff et al., 2008b). Such illusions depend on the expectations of the observers, and they can vary depending on whether they are frequently confronted with visual features such as right angles or vertical lines. For instance, one study found that Zulu children living in a traditional village with rounded huts were less prone to perceiving a rectangular shape in a visual illusion than Zulu children living in a Western-style city (Allport & Pettigrew, 1957). Perception can thus be distorted in the direction of familiar cultural features. Apart from effects on perception, culture and familiarity may also lead to differences in the preference for certain shapes or organization of shapes. For example, one study concluded that Aboriginal Australians may have an unusual preference for asymmetrical over symmetrical patterns (Bryers, 1976).

The effect of expertise with manipulation of abstract shapes is more difficult to quantify. Geometric shapes do occur in the environment even of unschooled children (even shapes that are rare in nature occur on man-made artifacts such as roadsigns), including in traditional craft (e.g., Gerdes, 1988), but these may not provide a sound basis for abstract manipulation, unless they are explicitly named and recognized as such (for an example, see Soares, 2009). It is worth recalling here that some children that have mastered mathematic operations on concrete objects are incapable of performing the same operations with abstract quantities (Nunes Carraher et al., 1985, 1987); likewise, it can be expected that subjects perfectly capable of manipulating concrete objects would have difficulties reasoning with abstract shapes. There may also be an effect of expertise on working memory load: in general, the ease with which information can be maintained in memory depends on expertise in manipulating it (Chase & Simon, 1973), so it can be expected that maintaining abstract shapes would be more cognitively demanding for subjects that are not used to working with them.

#### 4.2 Colors

The questions regarding cultural relativity of perception and categorization of colors are largely the same as for shapes. The same arguments regarding the Sapir-Whorf hypothesis of linguistic relativity have been raised and the same debates have occurred in the literature. Overall, there seem to be three relatively universal color distinctions in all languages (see Kay & Maffi, 1999, for an update of the classic Berlin & Kay, 1969 results): black versus white; warm (red and yellow) versus cool (blue and green); and red. It follows that for speakers of some languages, blue and green for example belong to the same taxonomic category. Even if they can be distinguished perceptually, they are not named separately, and they could be considered interchangeable in a visual task.

Although the first experiments failed to find an effect of availability of color names on cognition (Rosch Heider, 1972), subsequent research made it clear that cognitive representation and manipulation of colors is indeed influenced by whether a name is available or not (Lucy & Shweder, 1979, 1988), in direct relation with the use of verbal coding (Lucy & Shweder, 1988; Roberson & Davidoff, 2000). For example, languages that have two distinct terms for two colors exaggerate the subjective distance between the two (see Kay & Kempton, 1984, for a comparison between English and Tarahumara, an Uto-Aztecan language spoken in Mexico). A study with speakers of the Berinmo language of Papua New Guinea (which has five color words, for white, black, red, warm colors and cool colors) found that colors were perceived and remembered as a function of which color categories had a name (Roberson et al., 2000; see also Kay & Regier, 2007). Similar results were obtained with speakers of Himba in Namibia (Roberson et al., 2005). Another study found that speakers of Spanish and speakers of Yucatec, a Mayan language, had better memory for the colors that were also easier to communicate in their respective languages (Stefflre et al., 1966). Overall, the implications are similar to those for geometric shapes: colors that have a name will be easier to perceive, remember and manipulate.

#### 4.3 Numerosity

Although at the limit of what can be considered "visuo-spatial processing," the treatment of numerosity in a visual scene deserves a brief mention here because numerosity often appears in the construction of logical rules in visuo-spatial tests. For example, a subject may be required to understand that the amount of objects increases—one, two, three—in successive pictures. Some languages have an easier time expressing numeral concepts than others, and the complexity of numeric systems affects the speed with which they are learned (for a discussion, see e.g., Hunt & Agnoli, 1991). Some languages may count in base two, three or four (Gordon, 2004), which could affect mental representation of numerosity and what is considered a "complete" sequence. And some languages famously have only a few words for numbers. A classic example is the Pirahã language in Amazonia, which has been variously described as having words only for "one," "two" and "many" (Gordon, 2004; see also Nevins et al., 2009), or as having no words for numbers at all (Everett, 2005). Speakers of Pirahã have very poor performance in visuo-spatial tasks involving numerosity, such as perceptual matching of quantities of objects, or visuo-spatial memory (Gordon, 2004; see also Everett, 2005) and appear incapable of performing additions such as 1 + 1 (Everett, 2005). Pirahã is not quite an exception: there are at least a dozen other documented languages and language groups in the Amazon with no words for numbers above "one" (see Nevins et al., 2009).

These examples are extreme cases. Other languages lack terms for large numerals, which affects estimation of large numbers (e.g., Cole et al., 1974), but this is unlikely to play a role in most visuo-spatial reasoning tests. Another factor that could play a more important role than the availability of words, however, is attention to numerosity: Heron & Simonsson (1974) write about Zambia that "questions of amount or quantity are not dealt with in the terms of precision and exactitude with which they are invested in other cultures; the probability of a Zambian preschool child becoming aware of any importance being attached by his elders to exact identity or equivalence is effectively zero." And of course, there is the obvious issue of schooling (e.g., Nunes Carraher et al., 1985, 1987). Unschooled testees may be less oriented toward counting the precise number of elements in a visuo-spatial display.

#### 4.4 Size and distance

To my knowledge, there has been little study of cross-cultural differences in the perception of size and distance, but several authors have noted that some cultural groups lack detailed measurement units, or the willingness to employ them. As an example, Littlejohn (1963) provided a description of distance conceptualization in the Temne ethnic group in Sierra Leone. The Temne used time estimates such as "a day's journey" for very long distances, "the interval between two villages" for long distances (this is very approximate given that villages are not evenly spaced), "an earshot" for shorter distances. For everyday measurement, the Temne used a mix of various units such as "the outstretched arms of an adult man," or "the pace, the foot, the span, and lengths between knuckles in the forefingers." None of these units was precisely defined and they could not be converted into each other. Precise measurement, such as in hut building, was achieved by comparing objects with a model produced for the occasion. Areas could not be measured at all.

Cole et al. (1974) provided a similar account of measurement in the Kpelle group in Liberia. Long distances were usually given as subjective estimates ("not far"), or time estimates ("a walk of this many hours"). A Kpelle graduate student working with the authors expressed a distance as "four feet" in English but was incapable of finding a way to express the same thing in Kpelle: there was no well-defined measurement system for short distances. The authors commented that similar findings were obtained in Saulteaux Amerindians in Canada. An experiment on estimation showed that the Kpelle were liable to grossly misestimate short lengths (expressed in handspans, armspans or footlengths), and that their estimations with various units were not always consistent.

These cultural specificities might generalize to difficulties with length estimation in visuo-spatial materials, given that the measurement system being used can sometimes affect the precision of length estimates (e.g., Delgado, 2013). Critically, and as was the case for numerosity, the question of whether distance units exist at all is complicated by the question of whether distance is perceived as meaningful by the subject (Heron & Simonsson, 1974): some cultures place so little emphasis on exact measurement that differences of size may be viewed as completely irrelevant to the task at hand (e.g., Ombredane, 1951).

### 5 Encoding of spatial relations

#### 5.1 Use of a relative frame of reference

Visuo-spatial displays, especially the abstract displays used for intelligence tests, rely on relations between objects: for example, a square may be on the left side of a triangle in one scene, and on the right side in another. However, the system of encoding position relative to the speaker using terms such as "left" and "right" is not the only solution: another way to encode position is to refer to absolute directions, such as the cardinal "west" and "east." Surprisingly, the use of a relative frame of reference is not universal. Some languages, such as English, use a combination of relative and absolute systems; some languages exclusively use a relative system; and some languages do not use a relative system at all (for a detailed discussion, see Levinson, 1996, 1997a, b).

Such languages have no words for left, right, front or back, that could be used to describe the relation between two objects. Examples include aboriginal languages in Australia (Levinson, 1997a), such as Arrernte (Pederson et al., 1998) and Guugu Yimithirr (Haviland, 1993; Levinson, 1996, 1997b), some Tamil communities in India (Pederson, 1995), and Tzeltal, a Mayan language spoken in Mexico (Brown & Levinson, 1992). These languages fully replace relative with absolute directions: a square is not on the left side of a triangle, it is north of the triangle. Absolute directions can be cardinal points, in approximate reference to sun movements, but Tzeltal, which is spoken in a mountainous area, also uses absolute directions in terms of uphill/downhill (Brown & Levinson, 1993). Levinson (1996) describes a few other examples such as a mountain/sea axis in Austronesian languages (see also Dasen, 2018).

Note that this difference is not just a matter of expression: it seems to directly affect cognitive representation of space. Speakers of these languages mentally represent visual scenes in terms of absolute directions. Scenes are remembered and described in terms of absolute directions (Haviland, 1993; Levinson et al., 2002; Pederson et al., 1998): for example, when asked to describe the location of an object in a room 45 km away, a speaker of Guugu Yimithirr would say "it is over there" while making a gesture, and the observer would be expected to notice that the gesture was to the northeast, so that they knew to look in the northeast corner of the room (Levinson, 1997b). Hypotheticals and imaginary scenarios are also described in absolute terms: "go to the other side of the lake" is rendered as "go to the east side of the lake" (Levinson, 1997b). Tzeltal speakers remember and reproduce spatial arrays of objects as a function of the cardinal direction in which they were presented, not the orientation of the objects relative to themselves (Levinson, 1997b; Pederson et al., 1998; for a similar example with Tamil communities, see Pederson, 1995). This gives different results in the case where the speaker is rotated. Note that absolute and relative encoding are not informationally equivalent and cannot be reconstructed from each other: knowing that an object is to my left does not tell you how it is oriented in absolute terms, and vice versa (Levinson, 1997a).

The existence of potential effects on cognitive activity is clear. Multiple studies have provided evidence that the development of spatial language is causally related to the development of spatial cognition in children (e.g., Loewenstein & Gentner, 2005; Miller et al., 2017; Pruden et al., 2011) or even in deaf adults (Pyers et al., 2010). The precise effect on performance is more difficult to estimate. In some cases, it may be positive: speakers of Tzeltal or Guugu Yimithirr appear to permanently maintain a mental compass of their orientation relative to the cardinal points and a mental map of their surroundings. In surprise tests, they are able to indicate the angle between their position and a location hundreds of kilometers away "more or less at the speed of conversational response" and with very little error, even in closed rooms with no windows, a feat that would be difficult for Western readers to achieve (Levinson, 1996, 1997b).

On the other hand, in test contexts, forming a mental representation of abstract relations between objects in a visual display—at least as the Western observer perceives them—may be significantly complicated by the fact that a language lacks distinct words for directions relative to the observer. The ease of describing, chunking and mentally manipulating spatial descriptors depends on the mode of representation that is used (see also Levinson, 1997a). Describing or remembering relative spatial positions in Guugu Yimithiir requires information about the cardinal orientation of each object (instead of, or in addition to, an image of how they appear relative to the speaker), which could conceivably affect memory load or response speed.

Also problematic is that this makes the task dependent on how the item presented to the testee is physically oriented, relative to absolute directions. Cognitive description of an item (e.g., Figure 1) will be different depending on whether the paper is oriented north (the dot is to the north of the square); or west (the same dot is now to the west of the square). If the item is unaligned with a particular bearing, it will be more complex to encode the relative positions of figural elements, and this could hinder emergence of clear categorical representations (the dot is somewhere like 30 degrees north-east of the square). Moreover, some axes may not be equally polarized: Tzeltal speakers, who do not use relative coding, make a distinction between north, south, and "perpendicular to the north–south axis," which leads to more errors on spatial tasks that are oriented along the east–west axis (Levinson, 1997a). Rotations form another special case, given that their meaning is affected by the spatial coding scheme. For the testee, physically moving around a visual scene changes the orientation of the objects in a relative frame of reference, but not in absolute encoding; rotating the picture of test materials does not change the description of relations between objects in relative encoding, but it does in absolute terms (Levinson, 1996).

#### 5.2 Encoding of other relations between objects

Directions such as left and right are not the only ways to describe relational information in a picture: a language that does not have distinct names for prepositions such as *in*, *inside*, *among* and *between* may have difficulty accurately describing relations in an abstract visual scene designed by Western observers (Biesheuvel, 1951b).

Levinson (1996) provides multiple examples of such conceptual gaps in relational language: Guugu Yimithirr does not distinguish between "above" and "on," there is no word for "in," and the notion of "at" can be ambiguous. Korean lacks generic prepositions such as "on," "up," "down," "in" or "out" and can use distinct verbs depending on the object and the degree of fit (tight versus loose containment of an object by another; Choi & Bowerman, 1991). Similarly, Tzeltal does not have a single word for "in" or "on" and instead uses a range of words that depend on the precise shape of the object (Brown, 1994). Tzeltal also encodes the notions of "top," "front" and "back" in reference to shape and axial symmetry, so that a stone can have a "top" (which is dependent on its shape, but independent of its viewing angle), but a cube or a sphere cannot (Levinson, 1996). Obviously this can not only lead to more difficulty in describing a Western picture in the way intended by the designer: it can also lead to categorical errors if two different relations are designed by the same word—or if two relations conceptually identical to a Western observer are designed by different words.

### 6 Understanding representations of movement and time

#### 6.1 Movement and time in a single image

Movement is often represented in pictures, but perceiving movement in a static visual display is not straightforward. The first issue at play is the symbolic representation of movement within one picture, using conventions such as blurring, movement lines, or superposition of various states: these cues appear to be culture-bound and are not readily understood in all non-Western cultures. For example, one study found that less than half Bantu subjects in South Africa interpreted movement lines as intended, many perceiving them as a snake, or a trail of blood or water (Duncan et al., 1973, as reported in Deregowski, 1989; Serpell & Deregowski, 1980). Conversely, drawing a circle may signify a circular movement instead of a static abstract shape to some readers (Deregowski, 1989). A few cross-cultural studies suggested that African cultures have a greater tendency to perceive a single picture as a superposition of moments in time ("polyphasic perception": Wober, 1974; see Deregowski & Munro, 1974), though there has been little systematic evidence for this idea (see also Munro & Deregowski, 1976).

#### 6.2 Movement and time in a series of pictures

The other issue, more directly relevant to intelligence tests, is whether a succession of images (arranged as a line or as a matrix) is understood as a succession of states or moments in time. Deregowski (1989) noted that a succession of multiple images is not universally accepted as a representation of movement or successive states. Greenfield (1998) also highlighted that it is not straightforward to understand matrices as ordered sequences of pictures. In fact, and independently of order, it is not even straightforward to parse matrices as a structured set of pictures with a systematic organization in rows and columns (for a similar point, see Williams, 2013). Greenfield mentions one study showing that the use of matrix patterns for rug-weaving in Zinacante Mayans in Mexico is related to amount of schooling.

The matrix format is thus culture-bound, and there are a few cultural devices that can especially prepare observers to understand them. Greenfield (1998) insists that matrices in everyday life have become much more prominent in Western cultures, due to the use of technological media such as computers with spreadsheet softwares. Brouwer (1995) provides another example of a cultural artifact directly related to matrix problems: comic books, which are also arranged as left-to-right and top-to-bottom series of ordered pictures. Brouwer notes that untrained observers have trouble understanding such series of pictures at the temporal and causal level, frequently failing to recognize that the pictures depict the same thing at different moments in time.

It is worth recalling here that cultural biases in the horizontal direction of visual exploration can influence understanding of time and causal relations in linear displays: for example, observers have an easier time understanding visual scenes when they are temporally arranged in a direction that matches their reading system (i.e., with the subject on the left and the object on the right for left-to-right readers, and the other way around for right-to-left readers; Maass & Russo, 2003). The ability to understand the flow of series of pictures is thus related to literacy. For illiterate subjects parsing visual scenes with a strict structure, as in the case of visual matrices, understanding the causal connection between adjacent pictures could be more difficult.

### 7 Understanding three-dimensional representations

A significant amount of cross-cultural work has been dedicated to differences in the perception of the third dimension in visuo-spatial materials. I give only a brief summary here, because this topic is of secondary interest for visuo-spatial intelligence tests, which usually rely on abstract two-dimensional stimuli—although representation of three-dimensional objects is sometimes required for tests such as picture completion and object assembly. Detailed treatments of this question are given in Miller (1973), Serpell & Deregowski, (1980), and Deregowski (1989).

In a nutshell, understanding two-dimensional pictures as representations of three-dimensional objects or scenes requires specialized skills to correctly interpret an array of perceptual cues. Many depth cues, such as relief shading, relative size, superposition, or linear perspective, are cultural conventions that need to be acquired (Miller, 1973). Knowledge of these conventions, and consequently perception of the third dimension, increases with exposure to pictures (Hudson, 1962); besides, some cultures may simply not expect pictures to have a three-dimensional meaning (Littlejohn, 1963, gives this observation for Bantus). Failure to recognize the third dimension can lead to failures in recognizing objects (see “Recognizing pictures as representations” section), but also to misinterpretation of complex scenes (such as objects that are placed behind characters being perceived as part of the characters; Miller, 1973; Serpell & Deregowski, 1980).

### 8 Symbolic meaning

Shapes and colors tend to have symbolic meanings, which do not cross cultures. For example, the color red has different symbolism in Western countries, where it serves as a sign of danger and interdiction, and in eastern Asian countries where it tends to carry a positive connotation of joy. This is presumably one reason why test designers tend to avoid symbols with a cultural meaning, and focus on abstract, neutral shapes and colors. But there is a catch: a visual feature that seems to carry no symbolic meaning for a test designer from one culture can evoke something in a testee from another. Item 25 in Raven's Colored Progressive Matrices may not evoke anything for children in most cultures, but it will immediately strike French pupils as the *fleur-de-lys* symbol of French monarchy. As noted above, some readers may perceive a circle as the indication of a circular movement (Deregowski, 1989). Spatial directions such as left and right do not carry much of a symbolic meaning in Western cultures, but they do in others (e.g., Littlejohn, 1963).

I know of no data that would help determine to what extent symbolic meaning in a test may or may not affect performance in a given culture. There are at least four conceivable effects on processing: a recognizable symbol may draw the observer's attention to a greater extent than the rest of the scene, influencing visual exploration (see “Visual exploration” section); it could be perceived as a singular gestalt and become more difficult to decompose into component elements (see “Differences in analytic visual processing” section); conversely, a nameable symbol could be easier to chunk and manipulate mentally (see “Perception and processing of visual objects” section and “Encoding of spatial relations” section); and lastly, a symbol that conveys the idea of movement or spatial transformations, such as an arrow for Western observers, could lead the subject to misinterpret logical relations between objects.

In fact, the biggest cultural variation related to visual symbols may not reside in the content of a test item at all, but rather in the test instructions and response format: drawing a cross to indicate that something is wrong and a V to indicate that something is right is an arbitrary cultural convention that does not exist in many African cultures, where such signs mean nothing at all (Brouwer, 1995). Understanding instructions given using these symbols is therefore not straightforward, and neither is getting into the habit of using them to signify one's answer on successive items, which could influence response speed (Biesheuvel, 1951b).

### 9 Response production

The focus of this review is on perception and processing of visuo-spatial information, but the list of cultural differences would be incomplete without at least mentioning a related topic: motor execution of the selected response. Many intelligence tests require subjects to reproduce patterns by assembling wooden blocks, foam triangles or jigsaw puzzles: these materials are unknown in many cultures (Serpell, 1974, 1979) and can baffle subjects entirely (Ardila & Moreno, 2001; Jahoda, 1956; McFie, 1961). An extensive familiarization phase is often required (Jahoda, 1956; McFie, 1961), but this cannot be expected to erase any cultural difference: Western children have had many hours of practice assembling blocks and puzzles (Biesheuvel, 1951a; McFie, 1961), and the more expertise with this medium, the faster and more accurately responses can be produced.

Visuo-spatial tests also often require a response in the form of writing a number or drawing a picture, a map, a path through a maze… the problem is similar and has long been recognized by testers: this format requires basic familiarity with the use of a pencil, and subjects are sometimes asked to provide written responses without having ever used a pen (Ardila & Moreno, 2001; Rosselli & Ardila, 2003; Serpell, 1974; Wicherts et al., 2010b). In a series of experiments, Serpell (1974, 1979) found that Zambian children who often sculpt with pliable wire had much better visuo-constructive performance with this medium than with drawing, and they also did substantially better than English children who had much less experience with this medium.

## Application to the resolution of visuo-spatial intelligence tests

Having outlined the major sources of cross-cultural differences in the processing of visuo-spatial materials (Table 1), the next step is to model their possible effects on intelligence tests. In this section, I focus on Raven's matrices, because their solution process has been most systematically studied in the literature. This discussion is also applicable to other analogy tests. A few guidelines for other types of tests are proposed in the last subsection.

### An account of the solution process of Raven's matrices

Carpenter et al. (1990) provided a description of the solution process of Raven's matrices that is still the most widely used (see also Mulholland et al., 1980; for an alternative reading, see Primi, 2002). They based their investigation on a combination of eye-tracking, verbal reports, and simulations. The resulting process can be summarized as follows (my breakdown of the steps slightly differs from that of Carpenter and colleagues, but the contents are identical). Note that "entry" refers to one of the nine cells of the matrix, "components" to the various figural elements that constitute an entry (such as shapes), and "attributes" to the perceptual features of each component (location, orientation, numerosity, texture, etc.). The process is summarized in Table 2.

1. Visual exploration of the item. Subjects typically use a highly structured scanpath to analyze an item. They start by looking at the top left entry of the matrix, then scan the entries of the first line, proceeding by pairwise comparisons. They then move from the top to the bottom. There are occasional lookbacks, but this general flow from left to right and from top to bottom is respected; the row-wise organization is particularly prevalent. Of note, subjects who visually explore the whole matrix (as opposed to focusing their gaze on only some entries, such as the last row) perform substantially higher (Vigneau et al., 2006). Carpenter et al. (1990) do not consider this visual exploration as a distinct part of the process, as it is interleaved with the cognitive operations that underlie the next steps, but it makes the present discussion easier.
2. Decomposition of the perceptual gestalt in an entry of the matrix. Entries in the matrix are complex combinations of components: figural elements such as shapes, textures, and in some versions, colors. Except for the very easiest problems in Raven's matrices, which can be solved with a simple perceptual algorithm corresponding to pattern completion (Hunt, 1974), the logical rules cannot be understood at the level of these complex gestalts: entries need to be broken down into their constituent components. Subjects in the study of Carpenter et al. (1990) achieve this by listing the figural elements in an entry at the symbolic level, using verbal descriptions: "squares," "lines," etc. This corresponds to the *stimulus description* step of Carpenter and colleagues. This decomposition is not quite straightforward (see the example in Fig. 1, where dots can be merged with shapes) and can be affected by the relative complexity of the components (see Roberts et al., 2000).
3. Encoding the attributes of each component. The various perceptual features of each component in an entry need to be listed to allow for comparison. This requires identifying the perceptual dimensions of each component, as indexed by their name; and describing these dimensions, in terms of shape, color or texture, numerosity, spatial location, spatial orientation, etc. A square needs to be defined as, for example, *tall* and *striped*, a line as *wobbly* and *diagonal*, dots as *three in number* and *on the top of the figure*. This also requires storing the corresponding attributes in working memory (Carpenter et al., 1990), which presumably contributes to the significant working memory requirements of the task (Mulholland et al., 1980).
4. Finding correspondences between the components in adjacent entries. To understand the multiple rules that connect adjacent entries, the subject needs to understand which components are connected by the same rule. The most simple heuristic is based on shape, so that, e.g., "the number of *squares* increases from one entry to the next" or "the *triangles* are rotated clockwise." However, not all rules are based on shape, and sometimes the components that are connected by the same rule are defined by other figural features, such as their orientation (e.g., "the number of vertical lines, whatever their shape, increases from one entry to the next"). Carpenter et al. (1990) provide multiple examples of this and make it clear that this step can be a considerable source of ambiguity (see also Meo et al., 2007; Primi, 2002; Roberts et al., 2000). At any rate, the basic heuristic used by subjects to match figures together is their name: figures that have the same name are grouped together (Carpenter et al., 1990).
5. Pairwise comparison of the attributes of components in adjacent entries. Once matching components have been identified, they need to be compared to determine which of their attributes are the same and which are different. For example, there may be two squares in adjacent entries, which may be in same location but change color. This comparison process requires a same/different judgment on each attribute of each component. It also requires active maintenance of the results of this comparison in working memory, contributing to the overall working memory load (in the words of Pellegrino & Glaser, 1980, "nonidentity transformations require separate placekeepers in working memory").
6. Rule induction. Once differences between the attributes of components have been identified, the subject can formulate the rule that governs these differences (which can also be described, equivalently, as the rule that allows for the transformation of one component into another; Mulholland et al., 1980). There are multiple possible rules, including constant in a row (but changing across rows), quantitative progression across a row, figure addition or subtraction, and distribution of values (three different possible values for an attribute, each of which appears once in each row and in each column). Some rules can be understood in multiple ways, so this taxonomy is not exhaustive: for example, other frequently mentioned rules are movement across the plane, rotation (see Fig. 1), and changes of shape, texture or size (Jacobs & Vandeventer, 1972). The act of induction itself can be viewed as the process of generating a hypothesis about a rule that could be apply, testing this rule on the available components, and repeating this process until a candidate rule is found that matches the problem (for excellent treatments of this topic, see Egan & Greeno, 1974; Simon & Lea, 1974).
  The step of rule induction includes an additional process of generalization to the second and third row. Rule induction needs to be performed for the first row, then the second row, and lastly the third row. For the second row, the rules induced on the first row need to be applied again on the entries of the second row to ensure that they are correct; for the third row, this is the preliminary to generating the missing entry. This requires an additional operation of matching the corresponding figures across rows, identical to step 4, so as to know which rules of the first row apply to which figures on the second and third row.
7. Response generation and selection. Once all rules have been identified, they can be applied to components in entries of the third row to generate the missing entry. This requires integration in working memory of all the attributes of each component, after application of the correct rules (see Gonthier & Roulin, 2019; Mulholland et al., 1980; Pellegrino & Glaser, 1980). Importantly, the fact that the subject has to select one among multiple responses (rather than actually constructing the missing entry directly) allows for the use of a response elimination strategy that bypasses most of the preceding steps (see e.g., Gonthier & Roulin, 2019; Vigneau et al., 2006): the subject can select the response that seems most relevant, without having actually understood the matrix. This can be based on either partial induction of the rules, or on simple perceptual matching or selection of the most salient entry. This helps explain why the relative saliency of distracters can impact performance in the task (see in particular Arendasy & Sommer, 2013; Jarosz & Wiley, 2012; see also Matzen et al., 2010).

**Table 2:** Steps of the solution process for Raven's matrices, and expected cultural differences in visuo-spatial processing

Steps of the solution process	Examples of possible cultural differences
1. Structured visual exploration	Lack of understanding or attention to the pictorial information (2.1.1, 2.1.2, 2.1.3) Reading the item in the wrong horizontal direction (2.2.1) Lack of structured exploration of the matrix format (2.6.2) Attention to the wrong salient features (2.2.2, 2.8)
2. Decomposing the gestalt of an entry into its component figural elements	Failure or unwillingness to decompose the gestalt (2.3.1, 2.3.2) Failure to recognize distinct features as distinct components (2.4.1, 2.4.2) Failure to recognize a collection of features as a singular component (2.4.1, 2.4.2)
3. Encoding the attributes of each component	Failure to encode attributes that have no distinct name (2.4.1, 2.4.2) or a low cultural weight (2.4.1, 2.4.3, 2.4.4) Difficulty in encoding spatial relations using an inappropriate coding scheme (2.5.1, 2.5.2) Increased working memory load for attributes that lack dedicated names and for relations encoded in a complex way (2.4 and 2.5)
4. Matching components of adjacent entries	Failure to match components that cannot be reconciled in the same conceptual category Incorrect matching of components that belong to the same conceptual category (2.4.1, 2.4.2)
5. Pairwise comparison of attributes for adjacent entries	Increased working memory load due to lack of concepts or experience, leading to the omission of some attributes (2.4, 2.5) Incorrect judgments of sameness between perceptually different attributes (2.3, 2.4, 2.5)
6. Rule induction	Failure to recognize rules based on numeric operations (2.4.3) Failure to recognize rules based on movement (2.6.2) Failure to correctly generalize the rules to other rows due to unstructured visual exploration (2.2) or difficulty understanding the matrix format (2.6.2)
7. Response generation and selection	Increased working memory load leading to the omission of some rules during response generation Allocation of attention to culturally salient distracters (2.2.2, 2.8) Difficulty with motor execution of written responses (2.9)

Numbers in parentheses in the rightmost column refer to section headings, as also listed in the leftmost column of Table 1

### Expected cultural differences

This account of the solution process for Raven's matrices suggests two comments. Firstly, the test is highly visuo-spatial and intricately tied with accurate processing of visuo-spatial features. Performance depends on being able to decompose a gestalt, identify geometric shapes, understand the relations between pictures, maintain visuo-spatial information in memory, etc. It is clear that there is room for significant variation at the perceptual stage. Various authors have provided accounts of the effect of perceptual complexity in Raven's matrices (see in particular Primi, 2002; Meo et al., 2007). This presumably explains the substantial correlation between Raven's matrices and tests of spatial ability (e.g., Schweizer et al., 2007). In short, this is not "a test of *g*": it is a "visuo-spatial test of *g*."

Secondly, and although this may not be immediately apparent with superficial examination, the test is also highly verbal—not in the sense that it taps into complex verbal abilities that could elicit individual differences in a Western sample, but in the sense that it heavily relies on verbally-defined taxonomic categories and spatial relations. Take the example of verbal report presented by Carpenter et al. (1990): "Okay, there's diamond, square, triangle and they each contain lines through them with different shadings going from vertical, horizontal, oblique, and the third one should be—Okay, it should be a square and should have the black line in them and the answer's 5." I suspect that readers attempting to solve the example item in Fig. 1 will be talking to themselves using words such as "top," "middle," "bottom," "add up," "square" or "circle"; at least, that is how I do it. In the model of Carpenter and colleagues, multiple steps of the solution process are approximated based on name: components are defined based on names, their attributes are indexed based on names, components of adjacent entries are matched as a function of their names, and their attributes are judged as same or different as a function of name-driven taxonomic categories. This use of language to solve the test can obviously lead to cross-cultural differences. There is little direct evidence of this in the literature, but an example is found in Irvine (1969), who filmed children in Central Africa completing Raven's matrices, and found that they demonstrated considerable subvocalization. When prompted by the experimenter, some children reported subvocalizing in English, and some in their native language; but most children used a mix of the two. Irvine concluded that variance in test scores was partly driven by verbal differences.

Based on the cultural differences in visuo-spatial processing reviewed in the preceding section, the expected cultural influence for each step of the solution process are as follows (summarized in Table 2):

1. Visual exploration of the item: this requires familiarity and appropriate interaction with the paper medium and not, for example, trying to eat it, or paying less attention to the visual information than to the sound the paper makes when crumpled (“Interaction with paper” section); it requires paying attention to the picture drawn on the paper rather than to the paper itself (“Attention to the correct aspects of the paper medium” section); and it requires the insight-driven notion of representation—understanding that the figural elements on the paper are not interesting in themselves, but that they are supposed to represent logical concepts (“Recognizing pictures as representations” section). Such difficulties may be rare in most contemporary cultures, but they could still be a challenge to testing in very remote locations (e.g., Ardila & Moreno, 2001), and testees in cultures who have less frequent contact with paper may be more distracted by surface features irrelevant to the task. Readers familiar with the printed format of Raven's matrices will also notice that there is a very large white edge around pictures, which can be very salient and attention-grabbing in some cultures (Segall et al., 1966; see Miller, 1973).
  More relevant to most modern samples, this step requires structured visual exploration, dominated by left-to-right, row-wise movement. It should be noted that items are often constructed with a logic that is more obvious in one direction than in the other, e.g., when the third column (on the right) is the sum of the first two columns (on the left and middle). Of course, this can interfere with the spontaneous horizontal bias of subjects (“Horizontal bias as a function of reading direction” section). A few authors have documented the difficulty or even complete failure of subjects accustomed to right-to-left reading and visual exploration to perform matrix-like problems when presented in the left-to-right direction (Piswanger, 1975, as reported by Sternberg & Rifkin, 1979; Sternberg, 1999; van de Vijver & Tanzer, 2004). This has prompted some testers to mirror-reverse the items for use in right-to-left languages (e.g., Abdel-Khalek & Raven, 2000).
  More generally, this step requires exploration that is structured left to right and top to bottom, as driven by an understanding of the systematic causal connections between adjacent entries, which requires an understanding of the matrix structure. As discussed previously (“Movement and time in a series of pictures” section), this is not a cultural universal. This type of structured analysis will be less automated in subjects unfamiliar with this mode of representation and illiterate subjects, who can struggle with keeping an ordered visual exploration. The attention of some subjects may also be captured by certain salient features, such as colors (“Other biases in visual exploration” section) or even culturally meaningful symbols (“Symbolic meaning” section), contributing to inhomogenous allocation of visual attention. It is worth noting here that subjects who explore the matrix more systematically also perform higher (Vigneau et al., 2006).
2. Decomposing the perceptual gestalt represented by an entry in the matrix: the difficulty of certain non-Western samples in decomposing gestalts (“Decomposition of visual gestalts” section), or at least their disinclination to doing so (“Conceptual or cognitive styles” section), has been abundantly documented and can obviously interfere with this step. Perceiving an entry as a coherent whole and failing to decompose it into its constituent components prevents rule induction altogether for all but the most simple, perceptual items (Hunt, 1974).
  A more subtle issue is that the decomposition of the gestalt constituted by an entry appears to proceed based on the names of component figures (Carpenter et al., 1990): I know that an entry is composed of a square and a circle because I have a name for squares and circles. For non-Western subjects following the same process, it is easy to see how language differences can interfere with this step: if two figures or two colors belong to the same category in my language, I may be more inclined to considering them as a single gestalt, even if I can see them as perceptually distinct (“Geometric shapes” section and “Colors” section). Conversely, if a component has no name in my language, it may be more difficult to treat it as a singular component rather than a collection of features (“Geometric shapes” section and “Colors” section). Note that a lack of expertise with the manipulation of an abstract component, even if it does in fact have a distinct name, may also prevent the subject from treating it as a singular, self-consistent object.
3. Encoding the attributes of each component: the issues are similar to the preceding step, with a few complications. Listing the perceptual features of a component requires that I have distinct taxonomic categories for those perceptual features (“Geometric shapes” section and “Colors” section). Apart from shape and color, the issue of spatial relations appears here: the attributes of a component include its location and its orientation relative to other components in the same entry. I have to encode the fact that the square is "on the left of the circle," which may be more difficult in languages that lack distinct words for left and right, or top and bottom as applied to abstract shapes (“Use of a relative frame of reference” section and “Encoding of other relations between objects” section). Other attributes can include numerosity (“Numerosity” section), but also the relative size or distance between components (“Size and distance” section). As we have seen, both notions can be more or less difficult in various languages, and furthermore, some cultures place so little emphasis on numerosity or size/distance that they can be treated as irrelevant. Also recall that some cultures may place less emphasis on shapes than other dimensions (“Other biases in visual exploration” section). All these differences could lead to subjects in some cultures failing to (correctly) encode some attributes of a component.
  Assuming that attributes are not missed and are appropriately encoded, a further issue is that these attributes will have to be stored in working memory to allow for comparison with other entries. As discussed above, working memory load at this step can be expected to be much greater in some cultures, contributing to difficulty. This is either because attributes have no dedicated name in a language, or because the subjects have little expertise in manipulating them. This conclusion holds for the identity of elements (“Geometric shapes” section, “Colors” section and “Numerosity” section), but also for their spatial relations, given that some coding schemes are more complex than others in a given situation, and require encoding as a function of absolute cardinal directions or the shape of components (“Use of a relative frame of reference” section, “Encoding of other relations between objects” section).
4. Finding correspondences between components in adjacent entries: as stressed by Carpenter et al. (1990), subjects seem to usually perform this step based on names. Components that have the same names are matched together, which raises the same issues detailed above regarding the borders of conceptual categories. Is a square, in any sense, "the same thing" as a circle in my culture? (“Geometric shapes” section, “Colors” section). A subject may erroneously match components that are actually independent because they belong to the same category in their language, or fail to match components whose identities cannot be reconciled within the same category.
5. Pairwise comparison of adjacent entries: the two aspects of this step are maintaining in working memory all the features of components in two adjacent entries, and making same/different judgments on each of them. Both issues have been detailed above. Maintenance in working memory can be affected by the availability of names and by a subject expertise, leading to difficulty for cultures that lack them.
  Same/different judgments are usually described as mainly perceptual (this is primarily a question of determining whether attributes are exactly identical visually or not), but such judgments are not completely independent on an observer's perception of categories. After all, Western testees do ignore some perceptual differences as irrelevant to comparison of adjacent entries—such as minor irregularities in the test material, or simply the absolute position of a component on the sheet. Perception of conceptual categories is itself dependent on language, and on the emphasis their culture places on a particular dimension (“Perception and processing of visual objects” section and “Encoding of spatial relations” section). This is also directly related to the issue of observers emphasizing holistic perception and considering visuo-spatial displays as identical, despite obvious perceptual differences, as long as global features are respected (“Decomposition of visual gestalts” section and “Conceptual or cognitive styles” section). In Raven's matrices, this could lead to erroneously considering two distinct features as identical, and thus missing an important aspect of the rule. Related examples include testees incorrectly stating that block designs with obvious perceptual differences are "the same" (e.g., Pontius, 1995), and the tendency in certain cultures to treat mirror images as identical (Danziger & Pederson, 1998; Pederson et al., 1998).
6. Rule induction: the step of rule induction is obviously dependent on correct performance in all previous steps, so that failure to match components and encode and compare attributes can lead to induction of the wrong rule. There are however a few cultural differences in processing that could specifically impact this step. One is that knowledge of some rules may not be quite universal, or at least not as universally obvious as desired. This is the case of the addition/subtraction rules in cultures that lack detailed numeral systems (“Numerosity” section). This is also the case for rules based on movement: subjects in some cultures failing to recognize adjacent entries as a temporal or causal series (“Movement and time in a series of pictures” section) could make such rules more difficult to infer. Different modes of coding for spatial relations could lead some observers to treat rotation rules differently (“Use of a relative frame of reference” section).
  Another possible issue concerns specifically the step of generalization of rules induced in the first row to the second and third rows, which can tap into structured visual exploration of the matrix: as discussed for the second step, this requires both appropriate allocation of visual attention (“Visual exploration” section), and understanding of the causal structure of a matrix format (“Movement and time in a series of pictures” section).
7. Response generation and selection: mental generation of the missing entry requires integration in working memory of all rules, as applied to all attributes of all components. This is demanding for all subjects but, as discussed above, working memory load will be significantly greater for subjects who are less familiar with some perceptual dimensions (“Geometric shapes” section, “Colors” section, “Numerosity” section, “Size and distance” section) or who use different modes of coding for spatial positions (“Use of a relative frame of reference” section and “Encoding of other relations between objects” section). This could contribute to production of the most common type of error in Raven's matrices: selection of a response that is almost correct but misses one or several rules or components.
  Selection of the correct response can be affected by salient distracters, which raises again the problem of visual search and of what is considered relevant in a given culture (“Attention to the correct aspects of the paper medium” section, “Horizontal bias as a function of reading direction” section, “Other biases in visual exploration” section and even, to an extent, “Symbolic meaning” section). Distracters with a salient perceptual feature, such as an eye-catching color (see Fig. 1) or a shape with symbolic meaning, may elicit more errors in certain cultural groups. Cross-cultural differences in preferential types of pattern completion, such as preference for symmetry or asymmetry, could also draw attention to some distracters; an example would be the tendency in groups of Aboriginal Australians to select a distracter that duplicates the entry above the target (Bryer, 1976).
  Actual motor execution of the response should not be too difficult in the case of Raven's matrices… unless subjects are asked to write their response with a pen, or to index the correct response with an Arabic numeral (as in the example verbal report given by Carpenter et al., 1990), in which case differential familiarity with the response modalities will come in play (“Response production” section). This can contribute to slowness in responding, which can increase cognitive fatigue or decrease performance if a timed version of the test is given.

### A brief generalization to other tests

Raven's matrices have been particularly studied in the cognitive literature. For most visuo-spatial intelligence tests, no accounts of the solution process are available, or they are substantially less detailed. This makes it more difficult to infer the role of basic cultural differences in manipulation of visuo-spatial materials. However, a few tentative guidelines can be proposed here.

1. Block design tests such as Kohs' blocks (as in Fig. 2) require interaction with the printed target design (see “Understanding and interacting with pictures” section), a structured visual exploration of the target design (“Visual exploration” section), and the ability to decompose the gestalt constituted by this design into component shapes or blocks (“Differences in analytic visual processing” section). This decomposition is dependent on correct categorization of color (“Colors” section; see also Pontius, 1989). The most efficient strategy for this task is the synthetic strategy: breaking down the target design into geometric shapes made up of multiple blocks (see Rozencwajg, 1991; Rozencwajg & Corroyer, 2002), which can depend on knowledge of geometric shapes (“Geometric shapes” section). Since the test is timed, greater working memory load due to unfamiliar color or shapes could impose more lookbacks to the target design and affect performance. Likewise, differences in encoding spatial relations between sub-components of the design could play a part, with more complex coding schemes being detrimental (“Encoding of other relations between objects” section). Understanding the conversion between two-dimensional and three-dimensional representations is a particular issue for this task (“Understanding three-dimensional representations” section; see Fig. 1), with some authors noting the difficulty of subjects in understanding the relevance of trying to reproduce a two-dimensional design without using the sides of three-dimensional blocks (Serpell & Deregowski, 1980). And obviously, a lack of cultural familiarity with the manipulation of wooden blocks or similar puzzle pieces will be detrimental to performance (“Response production” section).
2. The mazes tests used by certain authors (e.g., Ardila & Moreno, 2001; Biesheuvel, 1951a; Cryns, 1962; Nissen et al., 1935) require interaction with the paper medium (“Understanding and interacting with pictures” section), systematic visual exploration (“Visual exploration” section), and use of a pencil (“Response production” section). If subjects happened to use internal language to scaffold their performance in the task, the mode of spatial coding could conceivably play a part (“Encoding of other relations between objects” section). A more subtle issue, however, is that the subjects may fail to understand that a maze is an aerial-view representation of a map: drawn representations of maps are a cultural device that needs to be acquired (see for example Downs, 1989; see also “Recognizing pictures as representations” section).
3. The figure weights subtest of Wechsler scales, which requires subjects to determine which figures are needed to balance a scale by inferring their weights, relies on interaction with the paper medium (“Understanding and interacting with pictures” section), systematic visual exploration resembling that required for Raven's matrices (“Visual exploration” section), decomposition of the set of figures on a scale into component elements (“Differences in analytic visual processing” section), attention to shape and color dimensions to adequately discriminate the elements, plus maintenance of their identity in working memory (“Geometric shapes” section and “Colors” section), and attention to numerosity (“Numerosity” section). (Of course, this list is restricted to cross-cultural differences in visuo-spatial processing, and overlooks differences in things such as familiarity with scales as cultural artifacts.)
4. The visual puzzles subtest of Wechsler scales requires subjects to find which among several pieces are needed to reproduce a target figure. Apart from the usual requirements of interaction with the medium (“Understanding and interacting with pictures” section) and systematic visual exploration (“Visual exploration” section), this task may be extremely reliant on the ability to decompose (or recompose) a gestalt into sub-components (“Differences in analytic visual processing” section). Moreover, the target figure (and often the possible pieces too) is a "good shape" by Western standards, such as a rectangle or a circle; such shapes may not have a name or a special status in many cultures (“Geometric shapes” section), which could make them more difficult to reproduce mentally.

## Conclusion

A considerable corpus of literature has shown that there are multiple cultural influences on the perception, manipulation and conceptualization of visuo-spatial materials. This review has shown how these various cultural influences can affect each successive step of Raven's matrices, and more generally of visuo-spatial intelligence tests. In sum, it appears that these tests are definitely not "culture-free." It is a certainty that differences in performance between ethnic groups do exist (Wicherts et al., 2010a), but to directly accept them as a deficiency on the part of subjects whose culture has far less prepared them to interpret and manipulate this type of materials is definitely an instance of ethnocentrism (Berry et al., 2002). In fact, given the extent of cultural differences in visuo-spatial processing, it would be surprising if there were no differences of performance at all.

It should be noted here that the literature does not actually provide empirical information about the weight of these various cultural influences on performance in an intelligence test. How worrying is it that the subjects have no name in their language for the geometric shapes involved in the test, that they focus less on shapes than on colors, that they have never seen a matrix-like format in their life? We currently have no way to answer this question, and supporters of mostly-genetic and mostly-cultural positions will no doubt make different hypotheses. What we do have is on one hand accounts of the solution process of reasoning tests, and on the other hand a body of literature showing that various mechanisms that differ across cultures could plausibly affect all the steps of this process. This review attempted to bridge the conceptual gap between these two lines of research, but direct experimentation would be required to pinpoint the contribution of each mechanism.

Such an effort would be all the more complex that this contribution certainly varies between different ethnic groups. Critically, however, it can be done: the detailed account provided here of cultural differences as they relate to an intelligence test can serve to generate testable predictions for the specific stimuli used in Raven's matrices. To name a few: eye-tracking could reveal less structured visual exploration of a matrix for non-Western readers (Vigneau et al., 2006, provide an example of measurable index), or differential susceptibility to certain salient distracters (see Jarosz & Wiley, 2012); a simple naming task could reveal greater difficulty in listing all component shapes in a complex gestalt as represented by the entries of a matrix; working memory capacity for abstract shapes or colors could be lower; there could be differences in categorization tasks for components of the entries used in the matrices, and in same/different judgment tasks for their perceptual attributes. These simple examples illustrate direct ways to assess the culture-fairness of a visuo-spatial intelligence task.

A question related to the weight of cultural differences, and one that will be particularly interesting to many readers, is the extent to which these cultural differences can affect domestic studies: comparisons between ethnic groups within the same country. Could these cultural differences in visuo-spatial processing contribute to the performance gap between "Blacks" and "Whites" within the USA, for instance? Cultural differences can be expected to have a substantial effect in the many studies comparing different countries (e.g., Lynn & Vanhanen, 2002; Meisenberg, 2004; Templer & Stephens, 2014), but a relatively smaller effect for ethnic groups sharing the same language and the same country: after all, children of African descent in the modern USA have read matrix-like comic books and know what a triangle is.

As mentioned earlier, however, a critical point is that familiarity with the cultural processing of visuo-spatial materials is not an all-or-nothing problem: it exists on a continuum. The greater the expertise with a mode of representation, the greater the ease with which it can be manipulated (e.g., Chase & Simon, 1973). Illiterate (unschooled) subjects within the same cultural group can demonstrate impairments on visuo-spatial tests, sometimes to a greater extent than verbal tests, in a way that is reminiscent of the results obtained with remote cultural groups (e.g., Matute et al., 2012). Therefore the effect of all cultural variations in visuo-spatial processing reviewed here cannot be discounted altogether, even within the same country. Cross-cultural differences within the same country could be particularly expected in terms of attention to the appropriate aspects of the test (“Attention to the correct aspects of the paper medium” section), automatization of horizontal visual exploration (“Horizontal bias as a function of reading direction” section), attentional capture by irrelevant visual features (“Other biases in visual exploration” section), relative skill in decomposing gestalts (“Decomposition of visual gestalts” section), expertise with manipulation of abstract shapes (“Geometric shapes” section), relative importance given to exact quantification (“Numerosity” section and “Size and distance” section), familiarity with two-dimensional representations of time and movement (“Movement and time in a series of pictures” section), possibly the interpretation of symbolic meaning (“Symbolic meaning” section), and definitely the skill in response production such as writing (“Response production” section).

Despite the lack of data on the exact weight of such cultural differences in visuo-spatial intelligence tests, my personal conviction (as with many other authors, e.g., Greenfield, 1997; Helms, 1992; Sternberg, 2004) is that the demonstrable existence of cultural differences (including qualitative differences in cognitive styles), the certainty that they play a role, and the *possibility* that this role may be substantial, are enough to disqualify the conclusion that some ethnic groups are in any sense inferior to others. This works in both directions: one study found that the average Maori man performed almost one standard deviation above Western controls in a block design task, but this can be attributed to cultural familiarity with a similar art form (tukutuku panels) rather than an innate superiority in visuo-spatial reasoning (Ogden & McFarlane-Nathan, 1997). 

Given that the present review has focused on the very specific case of visuo-spatial processing, it is also worth recalling here the large number of other cultural biases that can plausibly contribute to performance differences between ethnic groups. The list includes method biases (such as a lack of cultural emphasis on speeded performance; Agranovich, 2011; Ardila & Moreno, 2001), situational biases (see Kamin, 2006; Sternberg, 2004, also provides the example of a Tanzanian building that collapsed during intelligence testing), social and affective biases (such as stereotype threat: Steele, 1997), and construct biases (such as subjects considering the Western "correct response" on a test to be foolish: Greenfield, 1997), among others. The influence of each of these biases may be small when considered in isolation, but what is their combined effect on performance? And this is not even considering confounded differences of education (e.g., Ardila, 2005) or health (Boivin & Giordani, 1993; children in some African samples almost systematically suffer from malnutrition and parasitic illnesses: Sternberg, 2004).

Given that cultural differences do exist, a natural question is how to modify the tests so that they become culture-fair. For example, adaptations of Raven's matrices in Arabic-speaking countries have on occasion used the strategy of reversing items so that they read from right to left (Abdel-Khalek & Raven, 2000). This is definitely a good idea. However, it can be seen from the “Application to the resolution of visuo-spatial intelligence tests” section that this is far from a complete treatment of cultural differences, as this is not sufficient to erase differences in familiarity with pictorial material, linearly structured material, or with the matrix format; this does nothing either to address the lack of name for certain geometric shapes or the lack of habit in decomposing and manipulating them. Some authors have advocated retesting subjects after extensive familiarization with the test format (e.g., Jahoda, 1956; Ombredane, 1951; Sternberg et al., 2002). This also seems like a step in the right direction, although a limited familiarization phase will not replace decades of schooling and habit with manipulating geometric shapes, and there is a risk of artificially inflating scores. Studies attempting to replace abstract geometric components by more life-like objects are an interesting option which would deserve more detailed evaluation (Raven & Raven, 2000; Richardson, 1991, 1996; Richardson & Webster, 1996; Roberts et al., 2000; for a similar idea, see Bryer, 1976). Based on the data reviewed here, testable predictions can be generated to guide adaptation (see also Fagan, 2000), but there is no guarantee that a sufficiently adapted test would resemble the original version enough to allow for direct comparison with Western samples.

Does the existence of cultural bias mean that there is no place for inter-group comparisons of performance on intelligence tests? Far from it. My conviction is that the problem does not lie in the attempt to compare ethnic or cultural groups in itself, but rather in the inappropriate focus on total scores. Again, this is not a recent epiphany. Irvine wrote in 1963 that " the days of differences in mean score being produced as evidence of inferiority of basic mental structure are numbered." It is somewhat disheartening to see that this prediction has not really come to pass over the past sixty years (Borsboom, 2006). As the literature should make clear—along with “Application to the resolution of visuo-spatial intelligence tests” section in the present review -, visuo-spatial reasoning tests are highly complex, multi-step affairs, and there is much more going on than a direct measurement of *g*. The total number of correct responses on a visuo-spatial test tells us nothing about the multiple processes that had to be engaged on each item in order to reach that score (Detterman, 1982; Kovacs & Conway, 2016).

Average differences in total scores between ethnic groups are thus of no interest whatsoever to the cognitive psychologist, but a more detailed analysis of what cognitive processes elicited these differences in the first place could be useful. In other words, knowing that group A has on average a lower total score than group B is not informative, but pinpointing that this is due to group A failing to decompose a gestalt or failing to engage in structured visual exploration has the potential to inform cognitive and differential psychology about the steps of the solution process, their relative importance and fragility, and their interplay. This would allow the field to move beyond systemic racism and to contribute usefully to the understanding of human intelligence. Given that differences in mean scores between ethnic groups will, in all likelihood, continue to appear in the literature in years to come, my hope is that the present review can contribute to contextualizing them by providing a conceptual framework to better pinpoint their mechanistic origin in visuo-spatial intelligence tests.

## Data Availability

Not applicable.
